# ADHD and Identity Formation: Adolescents’ Experiences From the Healthcare System and Peer Relationships

**DOI:** 10.1177/10870547251318484

**Published:** 2025-02-18

**Authors:** Matilda A. Frick, Anders Brandt, Sandra Hellund, Jan Grimell

**Affiliations:** 1 Stockholm University, Sweden; 2 Uppsala University, Sweden; 3 Umeå University, Sweden

**Keywords:** ADHD, identity, adolescence, qualitative interviews, thematic analysis

## Abstract

**Objective:**

ADHD is often a lifelong condition, and has grown increasingly prevalent over the past few decades. Adolescence is a period characterized by the quest to develop a coherent identity, yet relatively little research has examined the relationship between ADHD diagnosis and identity. The purpose of this study was to explore the importance of experiences of the healthcare system and of peer relationships in the identity formation of adolescents with ADHD.

**Method:**

This was investigated through semi-structured interviews with 10 adolescents (*n* = 8 females and 2 males) aged 15 to 18 years. An inductive thematic analysis was conducted within a narrative framework.

**Results:**

The results revealed seven main themes indicating that ADHD played a central role in the adolescents’ self-narratives: (1) limited effect of Child and Adolescent Psychiatry (CAP) contact on identity formation, (2) the ADHD concept as meaning-making, (3) heterogeneity of the role of medication on identity formation, (4) negotiation of identity in relation to others, (5) varying degrees of acceptance in different relationships, (6) perceiving oneself as a troublemaker, and (7) relationship difficulties.

**Conclusions:**

Overall, the diagnosis constituted an important narrative and *symptoms* of ADHD rather than the *label* of ADHD tended to be stigmatizing. Furthermore, medication affected identity, and some felt pressured to medicate to adhere to social norms. A common pattern was that adolescents set aside their individual identity in favor of their relational identity.

## Introduction

The prevalence of ADHD has skyrocketed in Sweden and globally. A recent overview estimates that 9% of adolescents in Stockholm, Sweden have an ADHD diagnosis (Bölte et al., 2020), compared to the expected worldwide prevalence of 5% to 7% (Polanczyk et al., 2007, 2014). The Swedish National Board of Social Affairs and Health estimates that up to 15% of adolescent males and 10% of adolescent females may be diagnosed with ADHD before the trend levels off (Socialstyrelsen, 2023). ADHD is characterized by persistent and debilitating symptoms of inattention, hyperactivity, and impulsivity (American Psychiatric Association, 2013). Many adolescents who struggle with ADHD need support from the healthcare system (i.e., Child and Adolescent Psychiatry [CAP]) to manage everyday life. A diagnosis of ADHD generally comes with benefits and costs. Benefits may include pharmacological or psychological treatment, whereas costs may include stigma and social exclusion (Hansson Halleröd et al., 2015).

The reasons for the increase in ADHD diagnoses are not fully mapped, but likely include better awareness of the condition and heightened academic demands (Bölte et al., 2020). Furthermore, the ADHD diagnosis could be a sought-after marker of identity (Nielsen, 2017), that may function as an explanation for an individual’s inabilities to live up to societal expectations such as succeeding in school and in social life.

It is still unclear how identity is affected by receiving an ADHD diagnosis (Eccleston et al., 2019). Jones and Hesse (2018) propose that the diagnosis can lead to a re-evaluation of past experiences that are incorporated into an individual’s narrative of themselves. For instance, the feeling of being different or not fitting in could be better understood after being diagnosed (Andersson Frondelius et al., 2019; Jones & Hesse, 2018). Furthermore, some of the young people in Jones and Hesse’s (2018) study described that the diagnosis defined them and was visible to others, which led them to give up looking for an inclusive community. In contrast, some of the symptoms were seen as important qualities that they themselves valued. Andersson Frondelius et al. (2019) further showed that beyond the reassuring aspect of being diagnosed, there was a reluctance to belong to a group that was previously looked down upon. The diagnosis affected their social life, and in many cases they only disclosed the diagnosis to trusted friends, out of fear of being bullied or judged as mentally ill (Andersson Frondelius et al., 2019).

A large proportion of adolescents with ADHD receive pharmacological treatments, although the effects are varying and often limited (Faraone et al., 2021). The often-lifelong duration of ADHD and ADHD treatment requires further examination of how individuals create meaning from such a diagnosis in their life story and identity formation.

### Conceptualizing Identity in the Context of ADHD

Identity formation involves an interactive process, where daily experiences shape identity (Branje, 2022). That is, self-image and identity are negotiated via relationships and in interactions with institutions such as schools and the healthcare system, and include the story of who one is, where one belongs, and how one relates to other people and the outside world (Cerulo, 1997). Constructive and healthy identity formation is central to health, well-being, and successful participation in society. Erikson’s early work on psychosocial development states that adolescence concerns the transition from identity confusion to identity synthesis (Erikson, 1968). Findings suggest that adolescent girls may experience more identity confusion and less identity synthesis compared to boys (Bogaerts et al., 2021). Furthermore, identity confusion was associated with higher levels of depressive symptoms and identity synthesis was associated with lower levels of depressive symptoms (Bogaerts et al., 2021), suggesting that psychiatric issues may be related to poorer identity formation. Relatedly, adolescents with ADHD may be at risk of less constructive identity formation, with negative implications for health and well-being (Jones & Hesse, 2018), which could lead to a lack of belongingness, individual suffering, and high societal costs.

Identity is suggested to include several layers, including individual, relational, and collective aspects (Vignoles et al., 2011). Individual identity concerns goals in life, values, beliefs, and one’s life story (i.e., one’s narrative about oneself). Furthermore, relational identity concerns relationships with other people; for example, how the individual defines and perceives one’s role as a student, partner, child, or friend (Vignoles et al., 2011). Collective identity is about identification with different groups and categorizations in society, such as family and friendship groups, but also larger groups that deal with nationality, ethnicity, gender, and religion (Vignoles et al., 2011); and identity shaped by belonging to a group with a disability or diagnosis (Howard, 2000). Relatedly, an ADHD diagnosis can constitute a technoscientific identity (Clarke et al., 2003). By incorporating biomedical language into their story about themselves, a person can see and explain themselves based on their diagnosis. For example, a person can develop an identity that takes advantage of the descriptions of ADHD and thus see themselves as “impulsive” or “hyperactive.” The degree of identification with a diagnosis and views of one’s own diagnosis may affect how an individual perceives his or her future opportunities.

Being diagnosed with ADHD may impact identity formation at individual, relational, and collective levels. A systematic review of adolescents’ experience of living with an ADHD diagnosis showed that the diagnosis had an impact on the emerging identity (Eccleston et al., 2019). The stigma literature has sought to identify whether it is the label ADHD itself or its associated behaviors (i.e., symptoms) that contribute to stigmatization. A recent review concluded that effects were diverse; the label ADHD either exacerbated, mitigated, or did not affect stigma (O’Connor et al., 2022). Possibly, decreased stigma over the last few decades could partly explain the increased diagnosis rates, as the benefits of seeking treatment may outweigh fears of negative labeling (Ohan et al., 2013). A study on identity formation in adults suggests that a diagnosis was deemed necessary to gain access to support and helpful in better understanding oneself and in fostering a sense of belonging (Young et al., 2019).

Based on previous research (e.g., Eccleston et al., 2019; Jones & Hesse, 2018), there are clear indications that an ADHD diagnosis affects adolescents’ identity formation and personal narratives. Views and attitudes change across place and time, which highlights the importance of studying current beliefs and experiences of people living with ADHD in different countries and cultures. In consideration of the high and growing prevalence of ADHD, it is imperative to map what it means to an individual to live with the condition, with the long-term goal of favoring constructive and healthy identity formation. The purpose of the current study was to investigate what characterizes identity formation in adolescents with an ADHD diagnosis with a specific focus on two central aspects that may impact identity formation: (1) experiences from CAP, and (2) relationships with other people.

## Method

A qualitative interview approach was deemed especially suitable for this study, given the limited research on identity using primary sources within the Swedish context. In qualitative interview research, the significance or richness of the findings is not determined by the quantity of participants, but rather by achieving saturation, which is a sample size sufficiently large enough to reveal meaningful variations in the data (Kvale & Brinkmann, 2009; Polkinghorne, 2005). The study was performed in accordance with the Declaration of Helsinki and approved by the Ethical review board in Sweden (Drn. 2023-06162-01).

### Participants and Recruitment

Ten adolescents (*n* = 8 females and *n* = 2 males) aged 15 to 18 years (*M*_age_ = 16.4 years; *SD* = 0.8 years) participated in the study. Six of the participants stated that they only had ADHD, while the remaining four participants had comorbid conditions such as autism, dyslexia, eating disorders, anxiety, depression, and/or Tourette’s syndrome. Comorbidity was accepted, as it was hard to recruit participants with only ADHD. Accepting comorbidity also makes the sample more ecologically valid, as comorbidity is very common (Reale et al., 2017). Participants had been living with their ADHD diagnosis for varying periods of time; from just under a year to having received it during early childhood. The majority had been prescribed ADHD medication (see Table 1 for descriptive statistics). All 10 participants were born in Sweden and spoke Swedish as their native tongue. The parents’ highest level of education ranged from primary school (*n* = 1), upper secondary school (*n* = 4), university (*n* = 4), and unknown (*n* = 1). The participants were recruited in the Stockholm area during the winter of 2024 through advertising at upper secondary schools and via Meta. Interested participants completed an online survey to examine criteria for inclusion (age 15–18 years and having lived with ADHD for at least 6 months) and exclusion (no severe depression and no ongoing substance abuse) criteria. Twenty individuals expressed interest, of which 10 later declined or were not considered suitable for participation due to inclusion and exclusion criteria. The participants received a voucher equivalent to 10 euros as a token of appreciation for their participation.

**Table 1.** Sample Characteristics.

Measure	Mean (*SD*)	Range	Yes (*n*; %)
Age at diagnosis	11.4 (4.0) years	4–17 years	
Years since diagnosis	5 (3.6) years	1–12 years	
ADHD medication			8; 80
ASRS-A	52.4 (10.5)		
MADRS-S	20.7 (7.7)		
AUDIT	2.3 (3.9)		

*Note.* ASRS-A = Adult ADHD Self-Report Scale Adolescent version; MADRS-S = Montgomery Åsberg Depression Rating Scale; AUDIT = Alcohol Use Disorders Identification Test.

See Table 1 for sample characteristics. A description of the rating scales used in the online survey is outlined below. The Adult ADHD Self-Report Scale Adolescent version (ASRS-A; Kessler et al., 2005) was used to examine participants’ levels of ADHD symptoms. Participants’ responses were above the suggested clinical cutoff for Swedish adolescents with ADHD (Olofsdotter et al., 2023). The Montgomery Åsberg Depression Rating Scale (MADRS-S; Montgomery & Asberg, 1979) was used to assess the participants’ level of depressive symptoms. The included participants had, on average, a moderate level of depression, and none were deemed to have severe depression. The Alcohol Use Disorders Identification Test (AUDIT; J. B.Saunders et al., 1993), was used to assess the participants’ alcohol consumption. All included participants fell below the Swedish National Board of Health and Welfare’s limit values for risky use of alcohol. The Drug Use Disorders Identification Test (DUDIT; Hildebrand, 2015) was used to exclude those who indicated an active use of narcotics.

### Procedure

Before the interview, participants were given written and oral information about the study and filled out a written consent form. The interviews were conducted according to a predetermined interview guide (see Supplemental Material). We designed the interview guide to pose various types of questions related to identity and to encourage the interviewees to make narrative identity claims. That is, the interviews were designed to encourage participants to share and articulate how their identities and experiences are shaped and occur in real-life experiences in different areas of their lives (school, diagnosis, healthcare, leisure activities, and relationship to others). In the first part of the interview, we asked them about the process of getting their diagnosis. In the rest of the interview, we did not ask specific questions about ADHD, but instead let the participants bring up the topic spontaneously. The questions were open and intended to examine the participants’ experience of themselves and their identity and whether they had experienced a need to adapt themselves or their identities in different areas of life, with questions such as: “*In relation to others, have you experienced becoming someone else other than who you want to be or feel that you are*?” and “*How have you handled this clash or gap*?”

The interviews were conducted in Swedish by the second and third authors at the Department of Psychology at Stockholm University and audio-recorded. The material is stored in accordance with Stockholm University rules for storage of raw data and will be stored for 10 years after the last published article. The interviews were transcribed using Amberscript (https://www.amberscript.com), and then reviewed manually for content correction and de-identification of participants. After interviewing 10 participants, we found that saturation was reached (Saunders et al., 2018). That is, the interviews were detailed and rich in content and further data collection was not deemed necessary.

### Theoretical Framework

The conducted thematic analysis is positioned within a narrative theoretical framework, meaning that the participants’ interview narratives align with the conceptualization of identity as storied identity claims about who they are as individuals (McAdams et al., 2001, 2006; Riessman, 2008). That is, the fundamental assumptions in identity research with a narrative approach are that people express who they are, where they belong, and their place in the world through narrated claims (McAdams et al., 2006). Creating identities is thus a narrative process that can, in various ways, be captured through empirical research (Riessman, 2008). At the most basic level, this might involve asking a person to tell you who they are (McAdams, 2001). Thus, the theoretical framework in which we situate the inductive thematic analysis reflects a narrative understanding of the meaning of identity (McAdams, 1997, 2001). Although our analysis is not strictly narrative, the theoretical framework is based on the idea that participants, through their responses, present stories about who they are and assert their identity through their narrated answers (McAdams et al., 2001, 2006; Riessman, 2008). A narrative understanding of the empirical concept of identity does not necessarily require a narrative analysis. The framework is sufficiently open to allow other types of analysis to be understood within the context of a narrative understanding of identity.

### Analysis

The analysis was based on an inductively driven thematic analysis method. Thematic analysis in psychology has been used as a way to identify, analyze, and report patterns and themes in verbal responses (Braun & Clarke, 2006). Thematic analysis is not rooted in a specific theory, and thus carries the advantage of being flexibly incorporated into different theoretical frameworks. We used an inductively data-driven analysis method, where we let patterns and themes emerge from the material, based on guidelines by Braun and Clarke (2006) to get a better and deeper understanding of the participants’ narrated experiences. Our analysis was inductive, meaning that each transcribed interview narrative was individually coded. This process generated plenty of inductive codes that were both individual-specific, but which also converged with other participants. The subsequent goal was to develop themes based on these individual codes, allowing us to organize and present both inductive and individual codes meaningfully. The individual codes are reflected in the themes, which serve as clusters or categories that order and group the codes from the participants’ interview narratives. Analyses were performed by the second and third authors, under the supervision of the first author.

**Figure 1.**
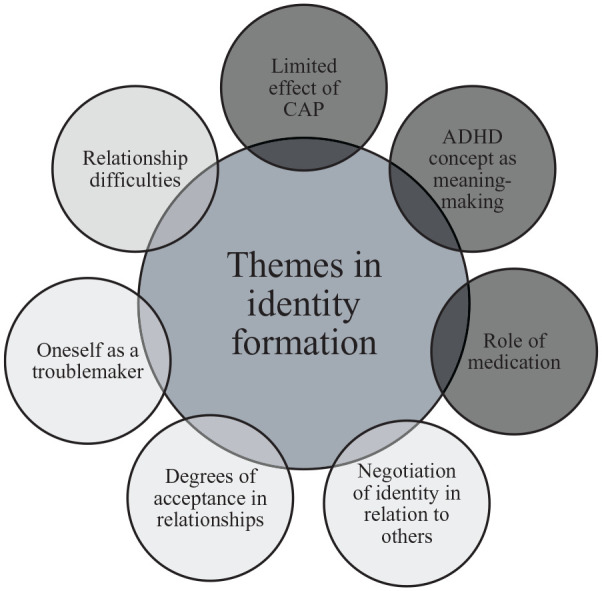
Depiction of the seven themes concerning identity formation in adolescents with ADHD. *Note.* Black circles refer to CAP-related themes and gray circles refer to themes related to relationships with other people. CAP = Child and Adolescent Psychiatry.

The thematic analysis consisted of the following six steps (Braun & Clarke, 2006):

1. We transcribed the interviews and read through the material several times to notice emerging codes and potential themes.
2. We individually created initial codes in a systematic way. All parts that related to the research questions were coded. Examples of codes include: “*diagnosis as explanatory*,” “*hyperactivity leads to conflict*,” and “*medication causes negative side effects*.”
3. Together we synced the individually assigned codes to form a common picture of the participants’ experiences. Through this, we obtained codes that were printed on pieces of paper (~600 pieces), which we grouped according to similarity. The process was reviewed, restructured, and reformulated several times and resulted in the development of themes.
4. We reviewed our themes again, to check that they formed coherent patterns and that they emerged in meaningful ways from the codes.
5. Once we had finally developed a thematic map of the material, we continued to define what was at the core of each theme. We then named the themes, which resulted in seven overarching themes (see Figure 1).
6. In the final step, we created the results section, selected quotes representing the themes and translated the selected quotes to English. Here, the narrative images of the participants were presented.

In the results section, the thematic analysis is presented, followed by narrative segments from participants that illustrate both the initial coding level and the contrasts and convergences between participants. In this way, the relationships between the higher thematic level and the codes are displayed.

## Results

The results section is organized as follows: (1) experiences from CAP, and (2) relationships with other people. See Figure 1 for an overview of the results.

### Identity Formation and Experiences From the CAP

Here, three themes emerged: limited effect of CAP contact on identity formation, the ADHD concept as meaning-making, and heterogeneity of the role of medication on identity formation.

#### Limited Effect of CAP Contact on Identity Formation

Contact with CAP had varying significance for the adolescents and how they viewed themselves after the clinical encounter with CAP. Three distinct experiential patterns were discerned. That is, the experiences of CAP could have a positive, mixed, or disappointing effect on participants, although most stated that CAP contact had not affected how they perceived themselves.

Some had had positive and supportive contacts with CAP: “*It has had a positive impact on me. That I knew that I could turn to them if there was something. If something bad had happened and I didn’t want to talk to anyone else.*” (Sophia)

To others, the experiences were more mixed in character: “*But I also have some trust issues from CAP because I feel that they promised a lot, but they didn’t give anything* [. . .] *The only time when I actually felt satisfied, it was with the psychologist I had who did the assessment on me.*” (Mia) Furthermore, “*I think it’s a bit complicated with CAP* [. . .] *I don’t really feel that I get a chance to talk to them because it’s like once every six months and then you have to bring everything up.*” (Alexander)

Yet others described disappointments, and that CAP contact had been substandard, lacking in what kind of help they offered or that the help came too late: “*I don’t think they helped. I’ve been to three different CAP things and in the last one I had, they said, ‘But we can’t help you.’ Then they sent me to intermediate care*.” (Denise) And “*But when I got the diagnoses then, it was like, ‘there are lots of courses we can offer here and to your parents’. But you weren’t allowed to go as soon as you had turned 18, and I turned 18 in October, so it was like we didn’t have time to take part in anything*” (Mia)

Furthermore, the assessment process and obtaining a diagnosis was not associated with stigma. Instead, it was seen as rather normalized to receive an ADHD diagnosis: “*I didn’t feel exactly abnormal. But like it was pretty accepted to have ADHD during that time*.” (Alexander)

In contrast, those individuals who had other diagnoses, such as autism or Tourette’s syndrome, described that these diagnoses were associated with a feeling of abnormality, and that the comorbid diagnoses had a greater negative impact on how they viewed themselves.

Mia had negative experiences regarding her contact with CAP. For her, it included a lack of effective help that only served to increase the mental problems for which she was seeking help. Mia was initially assessed for autism, but she received diagnoses for both ADHD and Asperger’s syndrome. Mia said it was shocking to receive the ADHD diagnosis as she was assessed for autism. At first, she did not recognize herself in ADHD, but she later came to think that the diagnosis reflected her way of being. She weighed the two diagnoses against each other and described how the autism diagnosis sometimes made her feel ashamed. She associated the diagnosis with a social stigma, and found it difficult to talk about the diagnosis: “*I’m a little ashamed sometimes when I have to say this autism* [. . .] *It feels like as soon as you see someone who is odd, they are like ‘it’s autistic.*’” The diagnoses held a multifaceted meaning in her story, which partly concerned how she viewed herself and was partly due to autism’s association with a higher degree of stigma compared to ADHD.

#### The ADHD Concept as Meaning-Making

A prominent theme was that although the adolescents perceived that *contact* with CAP had not affected their identity formation, the formal *diagnosis* clearly shaped the way the adolescents viewed themselves. They repeatedly referred to their ADHD diagnosis as a way of explaining themselves. The diagnosis was a way of creating meaning in their life experiences and hence formulating a meaningful story of who they are (i.e., a narrative identity). Most described how the diagnosis and the assessment process had led to a sense of relief at being able to explain their experiences and the many difficulties they had encountered: “*It’s nice that there was still a reason why I was pretty crazy. It’s like, ‘you’re not completely crazy. So, you’re like this and there’s nothing wrong with you*’” (Aida)

*“As soon as they started explaining what it meant and stuff, I kind of recognized myself, really on the spot. I was ‘That’s me, yes’. So now that I’ve read more and studied as well, I feel more familiar. I mean, I recognize myself more in ADHD than I do with the autism.”* (Mia)

*“It felt pretty good. I felt less of a failure. I feel more that I was good at other things than going to school.”* (Moa)

*“It felt like a relief to know that I had it and that I could get help. For me, it was very normal, but that’s how I wanted it on paper [. . .] I know there’s something about me, and it’s okay as well. I became a little more confident in myself and I knew that there was an explanation for why I was the way I was and that I could kind of fall back on it.”* (Sophia)

At the same time, the adolescents viewed their diagnosis in different ways, which also had implications for how they continued to narrate their identities in the aftermath of diagnosis. Some described ADHD as a disability, some as a disorder, others saw it more as a resource, and still others saw it as a part of themselves: “*ADHD is a superpower. I think so, but some people are like this that ‘it’s a disability’. But I really don’t think so*.” (Aida)

“*Well, I see it as a disorder.*” (Karen)

For some, accepting the diagnosis was a process that took some time: “*In the beginning it was very hard, in the way that I felt different. I felt very weird almost. It just didn’t feel like me and I felt like a different person. But now I don’t think so. First, because it is very common and there are a lot of people who relate.*” (Mia)

Aida saw the diagnosis in different ways. On the one hand, she described how the diagnosis was like a superpower and did not see it in a derogatory way. She did not necessarily see herself in terms of ADHD, but the diagnosis still held a central importance in her life and was something she had to navigate repeatedly in her identity formation. ADHD served as an explanation for her, and at the same time, she continuously thought about what was ADHD and what was her own person. Aida’s use of the ADHD concept showed that she extensively used it to explain parts of her functioning, and the way she was. For her, the diagnosis constituted a meaningful unit in her narrative.

#### Heterogeneity of the Role of Medication on Identity Formation

Virtually all adolescents provided recurring descriptions of being medicated for ADHD. We found a relatively large heterogeneity in how they experienced this, although most described that medication was CAP’s most commonly offered intervention, as Philip recounted: “*I have had contact with CAP since my ADHD diagnosis. But I didn’t have a psychologist, it was like ‘Okay, do you want medicine?’, ‘Yes’. Gave me medicine*.” (Philip)

For some, the medication enabled everyday function, which had an overall positive effect on their academic identity formation: “*But ADHD medication has done very, very much* [. . .] *I can concentrate on the lessons, and I can start a project. Not that I need to lie on the couch for 3 hours, before I stand up.*” (Moa)

Some stressed that medication was a way of not letting the diagnosis affect them: “*I would like to say that I don’t let my ADHD affect me directly, so much.* [. . .] *So I take pills now and I’ve been doing it for a while.*” (Alexander)

Others described direct negative effects of the medication, which also affected how they perceived themselves. Some felt compelled by the school or by their parents to medicate to ensure school functioning, and they needed to balance these demands with their own identity. The adolescents also described that the medication had helped in relationships, but sometimes dampened their emotional life, which had led to difficulties relating to others. Furthermore, for some the medication limited and dampened the experience of being themselves: “*The way I behave and move in everyday life, it changes a lot from when I take the pill and don’t take the tablet, so it makes me think ‘what would it have been like if I didn’t have ADHD?*’” (Karen)

In Karen’s story, we clearly saw how taking medication changed her experience of herself, which affected the individual and relational aspects of her identity. She expressed that she became a different person when she took medication; one which she did not like and one which her friends did not like, either. The medication affected her emotional life and limited her ability to relate to others. At the same time, she described perceiving pressure from school and from her parents to medicate, because it helped her cope with the school’s demands. She said that her non-medicated ADHD did not fit into the school, and that it was important for her to cope with school. She nuanced this picture by describing that the medicine made it possible for her to “*see reality from a normal person’s perspective*.” Karen described that she constantly had to deal with how the medication affected her and the demands the school placed on performance.

### Identity Formation and Relationships With Other People

Here, four themes emerged: negotiation of identity in relation to others, varying degrees of acceptance in different relationships, perceiving oneself as a troublemaker, and relationship difficulties.

#### Negotiation of Identity in Relation to Others

We found that the adolescents continuously negotiated their own identity, who they were in relation to others, and how they needed to adapt to others, which affected how they presented themselves to the social world. This negotiation seemed rooted in a common experience of feeling different. They repeatedly compared the image others had of them with how they viewed themselves. They often felt the need to inhibit themselves to fit in: “*And I guess I sometimes have to become someone I am not. Someone who is very calm and tries to really be quiet and not take up space. But, in turn, it makes me feel really bad*.” (Aida) And: “*But to protect myself, I have built up a wall of being a different person.*” (Sophia)

Similarly, some described that they imitated others to fit in: “*I try not to show that I have a diagnosis, so I kind of try to imitate them a little bit how they behave* [. . .] *when I meet my friends’ friends. And then new people that I haven’t met before.*” (Moa) Yet, others restrained themselves from spending time with others, so as not to get hurt: “*I don’t dare to be with people I might actually want to be with because I’m afraid it will be the same.*” (Sophia)

We also saw that the adolescents needed to navigate their own negative and positive perceptions of themselves. Negative beliefs often referred to a feeling of dissatisfaction with oneself and a lack of self-confidence. Positive beliefs, on the other hand, were about a belief in one’s ability and in being able to achieve personal goals. The navigation of identity in relation to others sometimes led to them changing social contexts, such as changing schools as a deliberate strategy to reshape the social image and identity that others had of them: “*I changed high school between 10*^th^ *and 11*^th^ *grade. And I’m really enjoying it now and I feel that I can really kind of be the person that I really am and want to be*.” (Mia)

Changing context led to an outlet for a more authentic version of oneself, which allowed them to construct identity in a desired manner. At the same time, for some participants, the process of navigating one’s own self included identification with people with similar difficulties who could act as positive role models. Identification with others functioned to normalize their condition and gave them hope: “*I have an uncle who also had an extremely tough time at school. But who is a very big role model. Okay, so I’m trying to imagine more that that’s the person I’m going to be*.” (Moa)

Alexander was dissatisfied with himself, felt insecure, and felt that he had a sense of inner obstacles that stood in his way. He described working on identifying change strategies; he wanted to look at himself in a more positive way, find more friends, and gain an increased belief in himself. Things that stood in the way were his feeling of being different and not fitting in, and that he often did not understand in the world around him, and did not feel accepted. He described that he did not feel that he reached his own goals or his potential and said that he had previously changed schools in order to be able to better show who he was in a new place and in a new context.

#### Varying Degrees of Acceptance in Different Relationships

The adolescents described how their relationships often involved varying degrees of how others accepted them. Family and close friendships were often characterized by high acceptance, support, and a sense of belonging: “*He really accepts me for who I am and it’s really nice and I can really just relax, just be myself. And he kind of doesn’t care if I get hyper because he gets hyper himself.*” (Mia)

This was contrasted by many other relationships, where participants were perceived as “difficult.” There was a constant navigation about how open they could be with their own identities; that is, how much they could really share with others. This contributed to an experience of not being accepted in many relationships.

“*Well, I don’t feel completely comfortable relaxing with the hyperactive because especially my mom bothers herself about it. We clash a lot there, that she is very opposite in it. Because she gets kind of stressed and then when I get hyper so like I stress her out then.*” (Mia) And: “*With girls, you can feel more of an openness than you do with guys like that.*” (Alexander)

Aida found acceptance both in individual friendships and in almost her entire circle of friends. She also talked about a teacher who allowed her to be exactly who she was. She described her family as accepting, but stated that she could not talk to her parents to the same extent as she had when she was younger. Aida described how she found an outlet for different parts of herself in different relationships, and that the different sides of her together made her feel like a whole person. When it came to how the outside world viewed her, Aida described that she had been told by adults from early childhood that she had difficulties, which led to her assessment during her preschool years. She said that she could be perceived as “hyper,” difficult, and stressful, which had led to her being judged, viewed negatively by others, and labeled as “bad.” She had encountered this from parents of her peers, from teachers at school, and sometimes from friends while growing up.

#### Perceiving Oneself as a Troublemaker

Here we found that the adolescents described how their own behaviors affected relationships and their identity. They often described everyday problems in terms of core symptoms of ADHD, such as hyperactivity, impulsivity, inattention, and forgetfulness, which had negative consequences for their interpersonal functioning. The adolescents saw ADHD as a contributing cause of difficulties in relationships and felt that they were not in control of their own behaviors. For example, this could manifest itself in participants ceasing to listen in the middle of conversations, which led to irritation from others; or impulsivity leading to ill-considered purchases, which led to conflicts in close relationships. This also had consequences both for how they viewed themselves and the identity characteristics attributed to them by other people in their social and relational proximities. These symptoms were described as obstacles in being able to become who they wanted to be. It was common for the adolescents to describe that fatigue and postponement of important tasks hindered their functioning, which led to failures or worry about future failures:

*“Then I also wish that I didn’t postpone things that I thought were difficult or that I wasn’t like this. Even though I really ‘But come on now, you have to do it’. But I kind of don’t.” (Aida) And: “I forgot to contact him, and I lost him completely. That’s kind of what I’m afraid will happen now, when I’m going to start in a completely new school. That I will be too inactive with people and lose contact.”* (Alexander)

Some described that the diagnosis was not stigmatized, but that the actual symptoms still led to problems: “*If people are like ‘Yes, but you have ADHD, that’s okay’, but then when I act like I have ADHD, then it can be like ‘God, it’s annoying.’*” (Mia)

Others experienced that the diagnosis turned them into someone they didn’t want to be: “*I felt unhappy with the person I had become, like that, said very controversial things* [. . .] *There was a period when I thought that at least half the class hates me, and I couldn’t stand it.*” (Alexander)

Alicia described how various difficulties linked to her ADHD diagnosis made her life difficult. She described how her problems with focusing on what was important led to her having difficulty living an independent life, and that her impulsivity led to spontaneous purchases, which in turn led to arguments with relatives. Furthermore, she said that others had become irritated when she could not concentrate on conversations she was in, which in turn led to her being annoyed with them. Alicia described that the problem of procrastinating and having difficulties getting started affected her negatively, as it was not in line with how she wanted to live and what she wanted to do with her life and who she wanted to be. It also led to her experiencing anxiety and not feeling that she could be the happy person she wanted to be, which affected her relationships.

#### Relationship Difficulties

The adolescents consistently described difficulties in relationships with peers, without clear reference to their own difficulties. This impacted their ability to engage in long-term, rich relationships, which in turn affected the formation of their relational identity. They described a lack of relationships and a desire to have more relationships. Recurring experiences included losing friends, being socially exposed, excluded, or bullied, and how these experiences led to a loss of trust in others. This led to changed attitudes toward other people: “*At the end of seventh grade, I gave up on making friends and just became the quiet one at the back of the classroom.*” (Moa)

*“When I was a kid, everything and everyone was good, I was running straight into everything and going for it. Then I’ve become more cautious lately, but I think it’s also very much about the people I’ve been around. I’ve become afraid of people as well. Because I’m afraid of getting hurt.”* (Mia)

*“And I feel that I’ve also been through a lot of people who aren’t very nice. And then I guess I’ve become more introverted and like my own company the most.”* (Sophia)

*“Yes, I have a very easy time falling into peer pressure and like I am very easily drawn to manipulative people, I very easily become this punching bag.”* (Mia)

Moa described how she wanted a higher level of social competence and to be more social. She described a close relationship as “insecure” and described how she had difficulty dealing with changes in large groups, such as school classes. She also described having recurring problems in her relationships with peers. In one case, these problems led to her giving up trying to make friends at school, and the difficulties in that particular relationship led to a conflict that ended the friendship. She said that this problem eventually led to her having to accept these relationship difficulties and move on with her life.

## Discussion

We set out to explore identity formation in adolescents with ADHD using qualitative interviews and inductive thematic analysis within a narrative framework. Our results propose that the ADHD diagnosis constituted an important narrative and that the *symptoms* of ADHD—rather than the *label* of ADHD—tended to be perceived as problematic by the adolescents and others. Further, medication affected participants’ identities, and some felt pressure to medicate to live up to academic expectations. Many made efforts to restrain themselves to fit in, resulting in a sense of not being themselves, setting aside their individual identity in favor of their relational identity. Here we discuss the takeaways of the study.

### The Importance of the Healthcare System in Identity Formation

The first takeaway revolves around CAP, as the adolescents in the study attributed a multifaceted meaning to CAP. The explicit impact of CAP on their identity formation was consistently described as small or non-existent, contrasting their statements that the formal ADHD diagnosis had led to an increased understanding of themselves, a sense of relief, and a way of creating meaning. As such, the diagnosis was central to the adolescents’ narratives about themselves, although this was largely seen as being separate from CAP. Hence, the diagnosis was not primarily seen as a medical label, but rather as a social label or category with which the adolescents identified. Their experienced deviations from generally accepted social and individual norms prompted an objective medical response; namely the assessment that led to the ADHD diagnosis. In turn, the diagnosis led to a new understanding of themselves and their past experiences, in line with findings by Jones and Hesse (2018). Furthermore, this new understanding of themselves seemed to progress from confusion to synthesis, corroborating Erikson’s groundbreaking work (Erikson, 1968). Indeed, in line with Erikson, early adolescence may be primarily associated with confusion, and later adolescence with synthesis (Bogaerts et al., 2021), which aligns with the narratives in our study. That is, participants described that they, over time, had progressed to a more coherent identity formation.

The ADHD diagnosis itself did not seem to constitute a stigma (Goffman, 1963) attributed by others or by themselves, although symptoms or associated behaviors could be perceived as stigmatizing or problematic by both parties. Indeed, comorbid diagnoses such as autism and Tourette’s syndrome were perceived as more stigmatizing than ADHD, and those with multiple diagnoses weighed the different diagnoses against each other. Previous literature points to stigmatizing attitudes toward people with ADHD (Lebowitz, 2016). However, the research we reviewed has mainly been conducted in countries other than Sweden, and is at least 5 to 10 years old, and attitudes toward diagnoses tend to change across time. We did not find that the participants held negative attitudes about the ADHD diagnosis per se, or that they encountered stigma from others to a great extent. The lack of stigma regarding the ADHD label could possibly be linked to the increase in diagnoses seen over the past few decades (Bölte et al., 2020). Self-identification with a diagnosis (even prior to assessment) can be a driving force, as the individual may be eager to get a diagnosis and associated treatment and therefore is more likely to seek care. However, we want to problematize this perspective, and focus on why an increasing number of children and adolescents pursue an ADHD diagnosis, interrogating also the structures and values of society.

A psychiatric diagnosis is a social and medical construct intended to manage what is deemed “deviant,” and such deviance is dependent on what is considered acceptable or unacceptable in society (Foucault, 1979). The Swedish school system, as a social context, has undergone significant changes over recent decades, including larger class sizes, and fewer personnel resources, along with overburdened teachers who are not only expected to teach but also to handle numerous other tasks unrelated to traditional teaching roles. In 2011, a major educational reform took place, including introducing grades earlier in the curriculum and national standardized tests starting in third grade. The reform decreased academic self-esteem and increased school-related stress, especially in girls (Högberg et al., 2021). In addition, Sweden is the country in the European Union with the largest proportion of failed pupils at the end of compulsory school, even though knowledge levels are similar (Gust et al., 2024), indicating that the educational grading system is to blame.

The changes may have led to the perception of who deviates academically becoming central at an earlier stage. In practice, the ability to play the role of a successful student—one who can sit still and learn—becomes crucial even before third grade, as the earlier years of education are oriented toward preparing for the national tests. Parents’ concerns should also be understood in relation to teachers who report that their child has difficulty sitting still. The step from such concerns to the thought “perhaps my child has ADHD,” is likely not a large one in a highly medicalized society (Conrad, 2005; Conrad et al., 2010), which tends to explain any deviation from the norm in terms of diagnoses (Foucault, 1979). Our discussion here is by no means exhaustive, but highlights that societal changes are essential to consider when examining the acceleration of ADHD diagnoses.

Medication played a central role in identity for many of the interviewed adolescents. Although perceptions of the effect differed, it was clear that they weighed the pros and cons of taking medication and how it affected identity formation. Some described that medication helped them to handle their diagnosis and fulfill academic expectations, whereas others had a feeling of not being oneself when medicated, and still others experienced social expectations to medicate. This is in line with Eccleston et al. (2019), who found that over half of the adolescents in their review reported a loss of identity or self-alienation as a result of medication. These aspects are central in shaping how medical treatments can be implemented to ensure individual autonomy. That is, adolescents, as they grow older, are allowed and encouraged to take part in decisions that concern his/her treatment. If the effect of pharmacological treatments is doubtful in many cases (Faraone et al., 2021), one must reflect on the effect of medication on teenagers’ identity formation.

Many of our results are in line with the results synthesized by Eccleston et al. (2019). We also found that the adolescents often were dissatisfied with the interventions offered by CAP. Similarly, the adolescents reported a lack of adequate psychosocial interventions (Eccleston et al., 2019). In all, the adolescents received mainly pharmacological treatments and the attitudes toward CAP were mainly negative. Our study thus contributes to the accumulated research that indicates that improvements are needed in the supportive efforts offered to people with ADHD. In addition to medical treatment, CAP could provide guidance to adolescents striving toward constructive identity formation, which with they currently seem to be left to their own devices.

### The Importance of Relationships in Identity Formation

Adolescence is a base camp for questions about self-image and identity. Indeed, the adolescents in our study dwelled on questions about their own identity and their ADHD diagnoses seemed to add a layer of complexity to the issue. We found that the adolescents wrestled with issues concerning individual and relational identity (Vignoles et al., 2011). In many cases, the adolescents described how they set aside their own individual identity in favor of creating or maintaining relationships with others. The participants further described how they constantly reflected on who they were in order to both be true to themselves and to fit in. When fitting in sometimes proved impossible, some changed social contexts or even schools. This constant reflection could be considered “ruminative exploration” (Luyckx et al., 2008), and may suggest that identity synthesis is not fully achieved. That is, although there are indications of identity synthesis, this may be delayed for adolescents with ADHD.

The participants described that in some close relationships with friends and family they were accepted and felt that they could be themselves to a greater extent. Although the diagnosis was not considered stigmatized per se, the adolescents described that sometimes others judged them for how they were. These stigmatizing attitudes are in line with what Martin et al. (2007) report about adults’ views of adolescents with ADHD and how many parents prefer that their children do not spend time with children with ADHD. This highlights a classic social anthropological perspective from Douglas (1966), which, in short, suggests that cultures tend to create purity through order. The preservation of order is a central driving force in cultures, which naturally have structures and functions to maintain it. What is chaotic and disorderly is considered impure and dangerous, something people seek to avoid due to fear that the disorder might spread to them. Douglas argued that the fear of contamination leads people to avoid others in a social sense to prevent contagion. While our culture, in some sense, creates order through diagnoses, chaos remains a form of disorder and danger, whose social contagion people may attempt to avoid.

In our study, it seems as if it was not the *label* ADHD that was stigmatized, but rather the behaviors and symptoms associated with the condition. The adolescents described how the symptoms of ADHD, such as impulsivity, difficulty maintaining focus, forgetfulness in staying in touch, and hyperactivity hindered their relationships and affected how they relate to other people. This means that symptoms stand in the way of many of the participants’ goals and aspirations. As such, it is the actual symptoms of ADHD, rather than the formal label of ADHD, that get in the way of their relationships and identity formation.

The adolescents negotiated different ways around issues of individual, relational, and collective identity (Vignoles et al., 2011). For example, we saw in Karen’s story that she was torn between meeting the school’s requirements by agreeing to medication for her ADHD and her own individual and relational identities. The consequence was that in the eyes of herself and her friends, she became someone else when she was medicated, while her collective identity as a productive student was maintained.

The adolescents described how difficulties in understanding and interpreting other people, together with difficulties in keeping in touch with friends, led to damaged friendships. They also described how they were exposed by peers and felt out-of-control in some relationships, which led the adolescents to lose trust in their peers, while they still longed for closeness. They described how they constantly needed to reflect on who they were in relation to others and that this was exhausting. They had mixed experiences of what perceptions they have about themselves. For example, a prominent way of dealing with a negative social image of themselves was to simply change the social context, which provided the benefits of being able to reshape oneself. In all, dealing with relationships and identity was clearly an area where the adolescents were in need of help and support. What this aid should constitute is a venue for future research.

### Limitations, Future Research, and Implications

The large majority of our participants were female, and as such, were not representative of the typical ADHD population. The female bias in qualitative ADHD studies has been noted elsewhere (e.g., Meyer et al., 2020), and gender differences regarding identity formation have been reported elsewhere (e.g., Bogaerts et al., 2021). We did not see clear differences in the participants’ answers based on gender, and none spontaneously reflected on gender during the interviews, but that does not rule out that other conclusions could have been made with more males in the sample. An avenue for future studies would be to address identity in relation to diagnosis and gender. Relatedly, our participants had had their diagnoses for different lengths of time. Although we did not examine the matter explicitly, there did not seem to be any differences regarding the value of the diagnosis, stigma, or any other aspects of the results that reflected time spent with the diagnosis. However, as identity formation is a continuous process, this matter should be investigated in future studies. Furthermore, we focused primarily on ADHD and our results suggest that ADHD was a central feature of identity. A different framing of the interviews may have contributed to a richer content of other identities, such as those related to sport or music. We address this as a limitation of our study.

Future research may examine how views on diagnoses have shifted in recent years, and what significance this has for identity formation and whether a decreased stigma regarding the diagnostic label of ADHD has contributed to the increase in prevalence in some countries (including Sweden). This highlights the importance of investigating the processes behind diagnostic self-identification, and how an individual creates meaning and integrates the diagnosis into his or her identity. Furthermore, findings should be quantified; for instance, it would be worth measuring how common it is to experience not feeling like oneself while on medication. Finally, there is a continued need to map out how to best support adolescents with ADHD in forming a positive identity and building stable relationships. We see value in adding an identity perspective when the effects of medication are evaluated. There may also be value in understanding the processes that have enabled social acceptance of ADHD to see whether it is possible to destigmatize other psychiatric diagnoses. However, one should keep in mind that the degree of stigma may be related to willingness to be diagnosed, and as such may increase prevalence. Contrasting individual interviews with focus group interviews may inspire emerging adults to create collective meaning that extends beyond what an individual informant may have access to. Finally, we see a need for both sociological and medical sociological perspectives to better understand how social and academic structures such as the grading system, large class sizes in schools, limited teacher resources, and increasing workloads beyond pure pedagogical concerns are related to the prevalence of ADHD and identity formation in adolescents with the condition.

As for implications, we see a need to offer support and targeted interventions to adolescents with ADHD to promote the development of constructive and healthy identity formation. Our results suggest that this work is currently done in solitude outside the healthcare system for many adolescents. The listeners’ responsiveness seems to be of essential importance in constructive identity formation (Pasupathi & Hoyt, 2009), and peers may not provide the encouragement needed by default. Group interventions that target identity may be a successful venue, as they can combine structure and guidance with much-needed social input from peers. Furthermore, structural interventions on a societal level such as in schools may be important to promote inclusion of diversity.

## Conclusions

In adolescents with ADHD, the diagnosis constituted an important narrative and undoubtedly affected identity formation. Symptoms of ADHD—rather than the label ADHD—tended to cause problems. Medication clearly impacted identity, but in different directions, and some felt pressure from teachers and parents to medicate. Many set aside their individual identity in favor of their relational identity. That is, they restrained themselves in order to fit in, with negative consequences for their sense of being true to themselves. Adolescents generally expressed that CAP had had very little impact on how they viewed themselves. At the same time, the ADHD diagnosis itself was meaning-making for them. This somewhat paradoxical finding is interesting, and enriches our understanding of ADHD. Our results seem to suggest that the way adolescents view ADHD diagnosis is disconnected from CAP; that is, adolescents see ADHD as a social category, rather than a medical one. In conclusion, the consequences of living with ADHD include social processes that are expressed in participants’ daily lives and affect identity formation.

## Supplemental Material

sj-docx-1-jad-10.1177_10870547251318484 – Supplemental material for ADHD and Identity Formation: Adolescents’ Experiences From the Healthcare System and Peer Relationships Supplemental material, sj-docx-1-jad-10.1177_10870547251318484 for ADHD and Identity Formation: Adolescents’ Experiences From the Healthcare System and Peer Relationships by Matilda A. Frick, Anders Brandt, Sandra Hellund and Jan Grimell in Journal of Attention Disorders
