# Supplementary material for: ADHD and Identity Formation: Adolescents’ Experiences From the Healthcare System and Peer Relationships
Source: J Atten Disord. 2025 Feb 18;29(7):541–53. doi: 10.1177/10870547251318484 (PMC11956369; doi:10.1177/10870547251318484)
Supplement: sj-docx-1-jad-10.1177_10870547251318484 – Supplemental material for ADHD and Identity Formation: Adolescents’ Experiences From the Healthcare System and Peer Relationships [file sj-docx-1-jad-10.1177_10870547251318484.docx]

# Interview guide

# Living with ADHD: Identity formation in adolescents with ADHD

## Introduction

- **Introduce yourself**

Name, who are you (master student), some general small talk to build an alliance and so that the person feels comfortable in the situation.

- **Tell the participant what will happen**

"Today I will interview you about your experiences and what it has been like for you to live with your ADHD diagnosis."

"We are doing this because we are investigating how young people experience what it is like to live with ADHD, so that society can provide the best possible care and support to young people with ADHD. That's why we're very happy that you want to join us and tell me about how it has been for you!"

"It is completely voluntary to do this interview. If there is a question that you do not want to answer, it is perfectly ok not to answer. You have the right to cancel the interview at any time without telling me why, and you can say at any time that you no longer want to be part of this study."

"I will record the audio as we talk and then the interviews will be written out in text. Audio and text files will be stored securely, so that no unauthorized persons can access them. The results will be presented in scientific articles. No personal information is reported there, so no one will be able to see what you have said."

"This interview will take about 1 to 1.5 hours. If you need a break, just let me know. As a thank you for being in the interview, you will receive a gift card."

"Do you have a question? Let's get started!"

[Make sure that written consent is obtained before you start the interview]

## Basic questions that explore narrative identity on a more general level from a contemporary perspective

1. Could you start by telling me a little about yourself. About who you are and what you like to do in your spare time?
2. Why do you think you're the way you are and why do you like these particular things?
3. If you think for a moment, is there a difference between who you feel you are and who you would like to be?

## Basic questions that explore narrative identity based on ADHD

1. You have an ADHD diagnosis. Can you tell me what it was like for you when you were diagnosed (how old were you, why was the investigation done, what did you think before, what was it like afterwards, how did it feel to get the diagnosis, did you tell the school/friends, how did it affect how you viewed yourself/how others viewed you)?
2. Have you received any special interventions in school or any treatment at the CAP based on your ADHD? Would you like to tell me a little more about them?
3. Is there anything that could have been done differently?

## Narrower questions that explore constructions of identities related to school

1. How do you feel your schooling has been? If that makes it easier, can you divide it into different periods (e.g. primary school, high school, etc.)?
2. During your schooling, have you experienced that you have become someone other than who you want to be or feel that you are?
3. Would you like to tell me a little more about this?
4. How have you handled this collision or gap?
5. What do you think could have been done to avoid this?
6. In what ways has it affected your relationships with classmates and teachers?

## Narrower questions that explore constructs of identities related to leisure activities

1. How do you feel that you have had it in your spare time and on any leisure activities? If it makes it easier, you can divide it into different periods or activities you have been in.
2. Have you in your spare time or during leisure activities experienced that you have become someone other than who you want to be or feel that you are?
3. Would you like to tell me a little more about this?
4. How have you handled this collision or gap?
5. What do you think could have been done to avoid this?
6. Are there contexts that you want to talk about where you have experienced that you are allowed to be exactly as you are or become who you want to be?
7. Would you like to tell me a little more about this?

## Narrower questions that explore the influence of healthcare on identity constructions

1. How do you feel that the contact with CAP has worked?
2. Would you like to tell me about how the contact with CAP has affected you?
3. Is there anything that could have been done differently?

## Narrower questions that explore constructions of identities in relation to other people

1. How do you feel that your relationships with other people have been? If it makes it easier, can you divide it into different periods (e.g. first years, high school etc.)?
2. Are there experiences that you in relation to others have become someone other than who you want to be or feel that you are?
3. Would you like to tell me a little more about this?
4. How have you handled this collision or gap?
5. What do you think could have been done to avoid this?
6. Are there other contexts that you would like to tell me about where you have experienced similar things?
7. How have you handled this collision or gap?
8. What do you think could have been done to avoid this?
9. Are there relationships where you have experienced that you are allowed to be exactly as you are or become who you want to be?
10. Would you like to tell me a little more about this?

## Final Questions

1. Is there any question I should have asked to understand you better?
2. Is there any question you would like to return to before we finish?
